# A modification of the reinforced Ross procedure: Root pressurization before implantation

**DOI:** 10.1016/j.xjtc.2025.03.017

**Published:** 2025-05-04

**Authors:** Gianna Dafflisio, Olivia McCloskey, Margaret Holland, Dominic P. Recco, Peter E. Hammer, Nathalie Roy, Michael H. Kwon, Akinobu Itoh, Sitaram M. Emani

**Affiliations:** a Department Cardiothoracic Surgery, Boston Children's Hospital, Boston, Mass; b Division of Cardiac Surgery, Department of Surgery, Brigham and Women's Hospital, Boston, Mass

**Figure:**
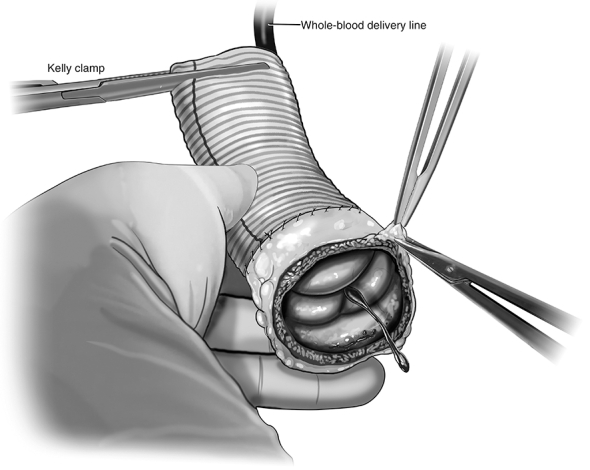
Ross autograft pressurization, assessment of function, and supportive procedures.

**Central Message**

Pressurization of the reinforced Ross autograft before implantation allows for assessment of autograft function and optimization of valve performance through individualized supportive procedures.

The Ross procedure is a widely accepted option to treat severe aortic valve disease, especially in younger adults. In recent years, the Ross has become increasingly used as the result of its favorable durability when compared with mechanical, bioprosthetic, and homograft valves^1^, ^2^, ^3^, ^4^, ^5^ and its avoidance of life-long anticoagulation. Despite these many benefits, the Ross is a technically difficult procedure, and there are several mechanisms by which it may have early and late failure.

Ross autograft late root dilation is a major issue that can necessitate autograft reoperation.^6^^,^^7^ Late root dilation has been addressed with techniques such as the inclusion technique, reinforcement of the root with a Dacron graft or other materials, or an autograft reduction plasty. These techniques have been successful and have significantly reduced autograft root dilation and dysfunction^8^, ^9^, ^10^, ^11^; however, none of these techniques assess autograft valve function before implantation.

We therefore developed a technique to (1) help identify patients for whom immediate Ross autograft regurgitation may occur and (2) support the reinforced autograft through individualized procedures aimed at optimizing the autograft's function in the short and long term. This technique consists of the pressurization of the Ross autograft before implantation. Herein, we describe the surgical technique and report the perioperative outcomes of the first 4 patients who underwent this modification of the reinforced Ross procedure. Data can be made available upon individual request to the corresponding author.

## Methods

### Study Design and Patients

The study was approved by Boston Children's Hospital Institutional Review Board (IRB-P00048572, approval January 14, 2025) with need for patient consent waived. A double-center, retrospective review was conducted of the first 4 patients who underwent a modified reinforced Ross procedure at Boston Children's Hospital and the Brigham and Women's Hospital between September 2024 and January 2025.

### Data Collection and Reporting

Patient demographics, perioperative imaging, operative details, and postoperative outcomes were collected from patient charts. Imaging data were sourced from echocardiograms and cardiac computed tomography scans and included pulmonary and aortic root measurements and valvular and ventricular function. Operative details included surgical technique, Dacron graft sizes, and procedural times. Postoperative outcomes included blood pressure, blood pressure management, intensive care unit (ICU) and hospital lengths of stay, major complications, reinterventions, and mortality. Measured values are summarized using median and range.

### Feasibility Studies

Initial feasibility studies were done in an ex vivo setting using expired pulmonary homografts and porcine hearts. Six models were completed to evaluate the logistics and efficacy of both the pressurization technique and the optimization procedures.

### Operative Technique

A full demonstration of the operative technique is shown in Video 1.

#### Autograft harvest

**Figure 1:**
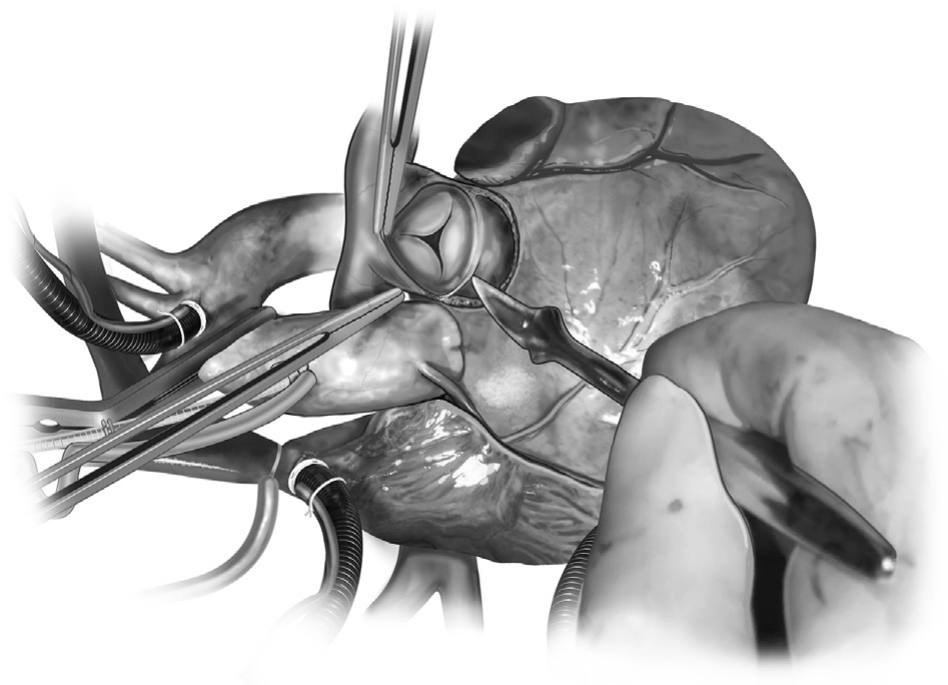
Autograft harvest. The right ventricular outflow tract incision is started 3 to 4 mm below the hinge points of the leaflet cusps. The first incision is made superficially and then skived up to avoid injury to the first septal perforator.

If there is no intra-atrial communication, harvesting of the pulmonary artery (PA) root can be done on bypass with beating heart. If there are intracardiac shunts, PA root harvest can either be done fibrillating or after aortic crossclamp. The pulmonary root is dissected from the aortic root, and the right ventricular outflow tract incision is started 3 to 4 mm below the hinge points of the leaflet cusps. The first incision is made superficially and then skived up to avoid injury to the first septal perforator (Figure 1).

#### Pressurization set-up

If the PA root harvest is performed with beating heart, the PA autograft can be prepared directly on the field while the heart remains perfused and beating on bypass. If done with an aortic crossclamp, autograft preparation can be done by a second operator at a back table to minimize ischemic time while the primary surgeon prepares the left ventricular outflow tract (LVOT) for autograft implantation.

Using the cardiopulmonary bypass (CPB) machine, an additional length of quarter-inch tubing is added to the cardioplegia line with a 3-way stopcock, allowing the perfusionist to administer pressure-targeted whole-blood flow to the autograft. The remaining components of the pressurization system are a straight or curved Kelly hemostatic forceps to prevent back flow, a large basin for fluid collection, and a drop suction to allow for recollection of blood.

#### Autograft pressurization and preparation

**Figure 2:**
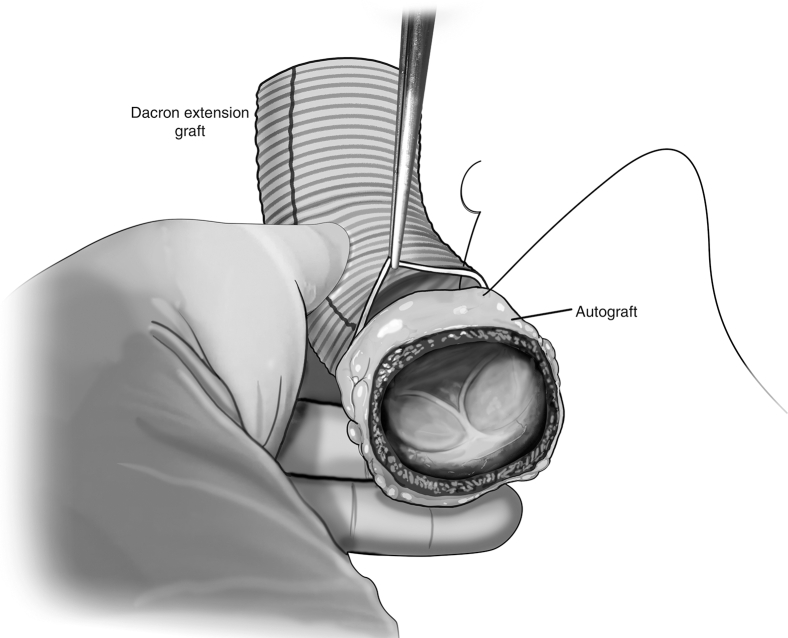
Distal extension of autograft. The distal end of the autograft is trimmed to leave 3 to 4 mm of pulmonary artery distal to the tops of the commissures. A Dacron tube graft is sewn to the distal end of the autograft using running 5-0 monofilament suture.

**Figure 3:**
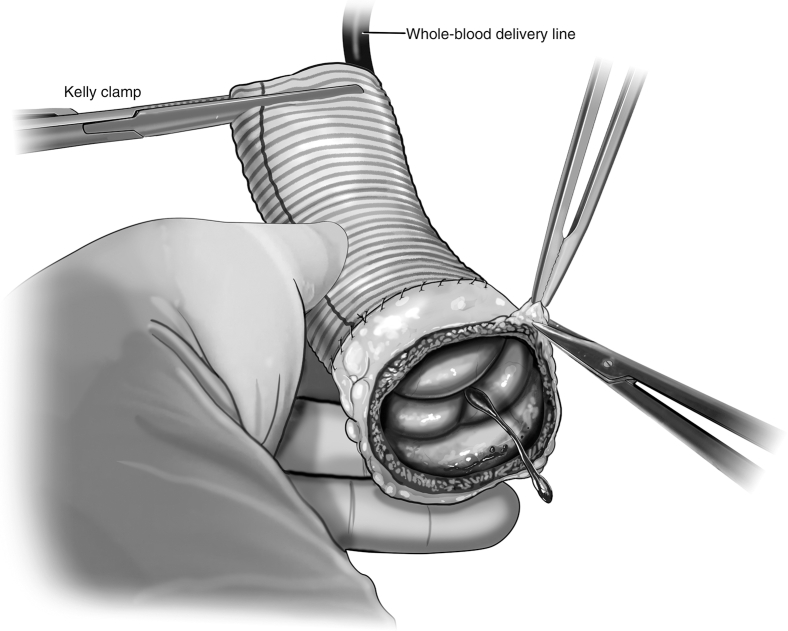
Autograft pressurization and assessment. Autograft pressurization is achieved by inserting a line from the cardiopulmonary bypass machine into the distal end of the Dacron extension graft. A clamp secures the graft around the tubing, and whole-blood is delivered to the autograft. This pressurizes the root, allowing for assessment of autograft function. Individualized procedures to optimize graft function such as adventitial trimming can then be done.

The autograft is inspected, and any evident excess tissue is trimmed from the muscular cuff and adventitial fat. The distal end of the autograft is trimmed to leave 3 to 4 mm of PA distal to the tops of the commissures. The autograft is extended distally with a Dacron tube graft using running 5-0 monofilament suture (Figure 2). The size of the ascending Dacron graft is chosen on the basis of preoperative measurement of the PA sinotubular junction diameter by cross-sectional imaging, in conjunction with intraoperative sizing of the PA with external sizers. Given that the pulmonary root can be somewhat elliptical in cross-section, an average of the diameters is used to select the ascending graft size. Then, the autograft is pressurized by inserting the additional tubing off of the cardioplegia line into the distal end of the Dacron graft and clamping the excess graft around the tube to prevent back-flow (Figure 3).

**Figure 4:**
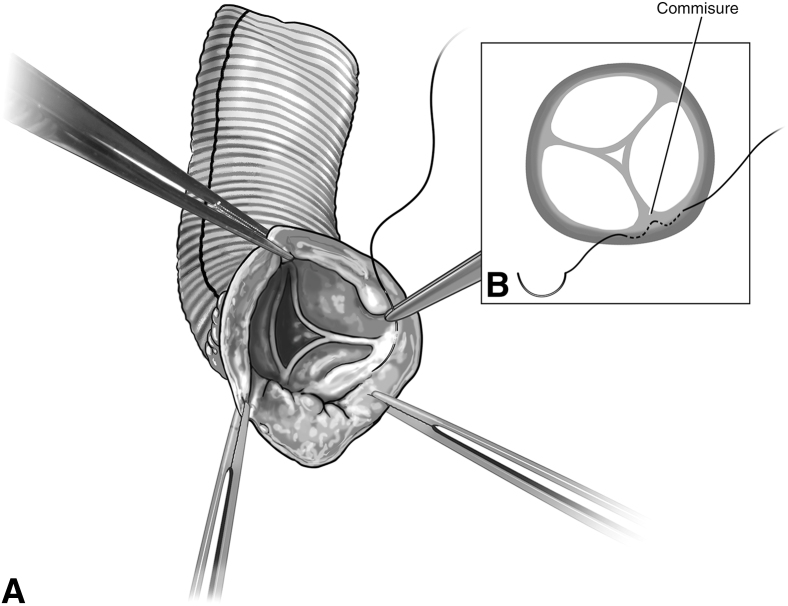
A, Placement of the subcommissural plication stitch. B, A subcommissural plication stitch can be placed while the autograft is depressurized to provide additional support in the case of noncentral regurgitation.

The perfusionist can then pressurize the graft using the cardioplegia delivery system, allowing for a sterile source of blood with flow control. The root is initially pressurized to 40 to 60 mm Hg to grossly assess autograft condition. The autograft leaflets are then inspected from the ventricular side for adequacy of coaptation and areas of regurgitation. There is often a trivial amount of regurgitation emanating centrally (Figure 3). Minimal central regurgitation does not purport autograft dysfunction. If a regurgitant jet appears at the commissures, a subcommissural plication stitch can be placed with the autograft in a depressurized state (Figure 4).

**Figure 5:**
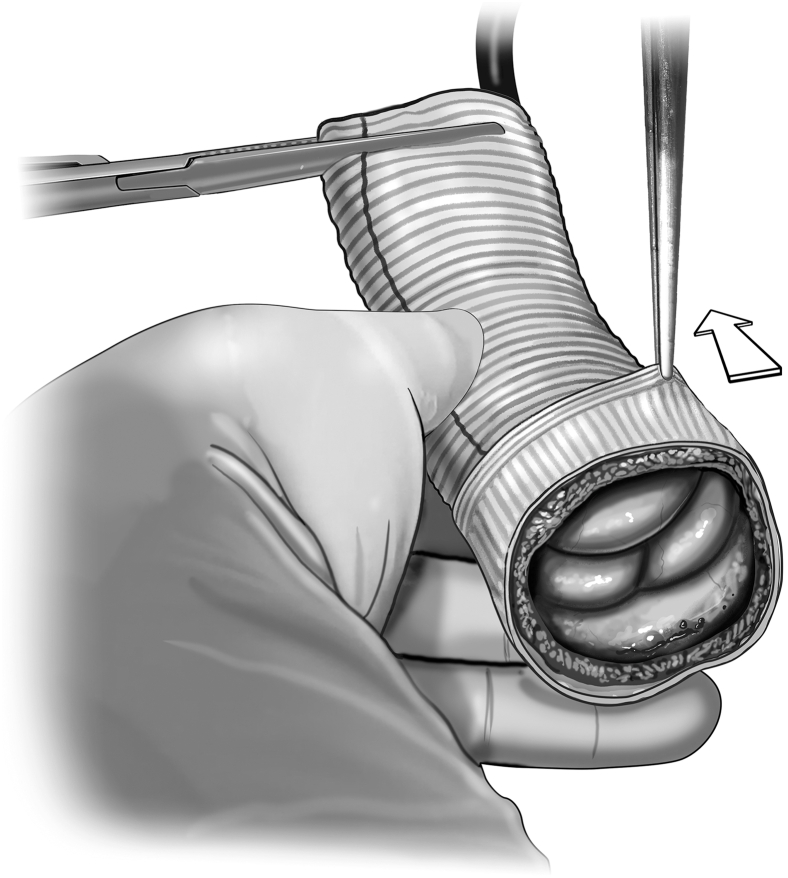
Placement of root reinforcing tube graft. A root reinforcing tube graft is placed around the autograft to assess initial fit. Additional adventitial or muscular cuff trimming can be performed to ensure the autograft is not crowded.

The autograft is placed within a root-reinforcing Dacron graft (Figure 5). We prefer a Valsalva graft (Terumo Aortic), but a straight graft can also be used. If using a Valsalva graft, the autograft sinuses are aligned within the graft skirt. The root graft size is determined by adding 4 mm to the preoperative measurement of the PA sinotubular junction diameter to accommodate autograft wall thickness. This typically translates into a root graft size that is 1 or 2 sizes greater than the ascending aorta graft. Attention is paid to the fit of the pressurized autograft within the Dacron root graft, as some level of reduction of the autograft is often needed. Additional adventitial fat can be trimmed, as can excess muscle once the cuff is visualized within the root graft. A large cuff is not necessary, only 2 to 3 mm of muscle proximal to the valve hinge points are needed to complete the LVOT anastomosis.

**Figure 6:**
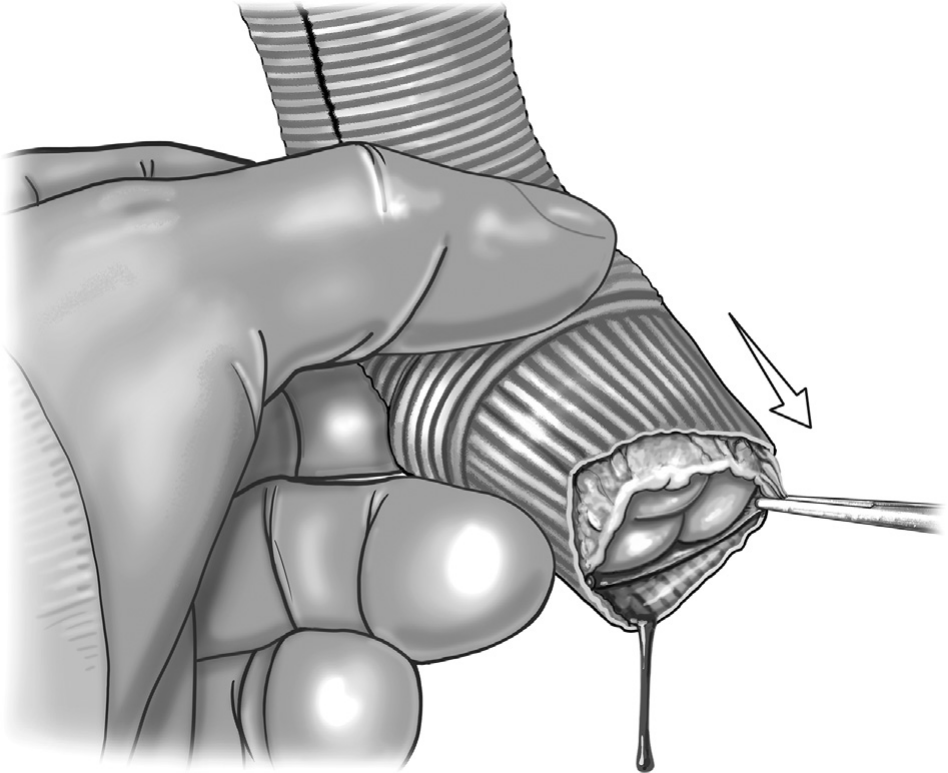
Adjustment of autograft orientation within root graft. While pressurized, the plane and height of the autograft are manipulated within the root graft to evaluate which orientation and location allows for optimal valve competency.

**Figure 7:**
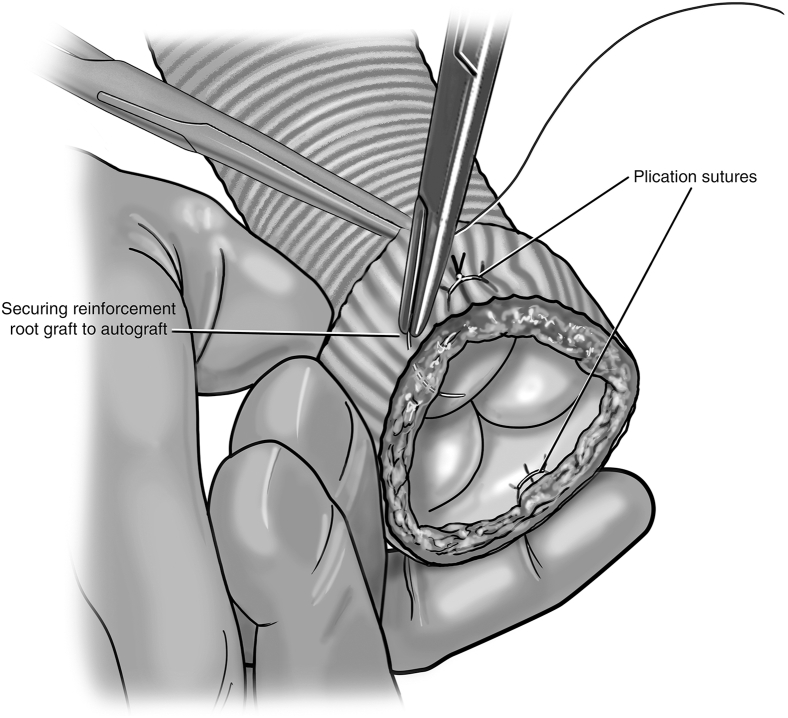
Annular plication and securing root graft to autograft. Full-thickness annular plication stitches can be placed if determined to reduce regurgitation. Once coaptation is optimized, the autograft remains pressurized and the proximal end of the autograft is secured to the root graft using interrupted sutures.

The autograft is then pressurized once more, with the root graft slipped on but not secured to the root. Pressurization is now performed to 60 to 80 mm Hg to evaluate autograft function at higher pressures. A series of maneuvers can optimize autograft function before stabilization of the root graft. The leaflets are again inspected from the ventricular side. This time, attention is paid to how and where the autograft sits within the Dacron root graft. Manipulations can be made to both the plane and height at which the valve lies in the root graft (Figure 6). Assessing for optimal valve competency during these maneuvers ensures proper alignment of the autograft commissures within the root graft. Trimming of the proximal end of the root graft may be done if Dacron protrudes past the autograft muscular cuff after finding the optimal placement. During this stage, one can also place subannular plication stitches in order to reduce the annular size of the autograft and/or root graft if determined to improve coaptation and reduce regurgitation (Figure 7). Once the optimal configuration of autograft seating within the root graft has been established, the root remains pressurized and the proximal end of the autograft is secured to the root graft using interrupted 5-0 monofilament sutures (Figure 7). A running suture can also be used. The distal end of the root graft is not secured to the ascending graft at this time in order to account for aortic curvature after distal anastomosis of the ascending graft. The valve commissures are then denoted with a sterile marking pen on the root graft, and the autograft can be depressurized.

#### Autograft implantation

**Figure 8:**
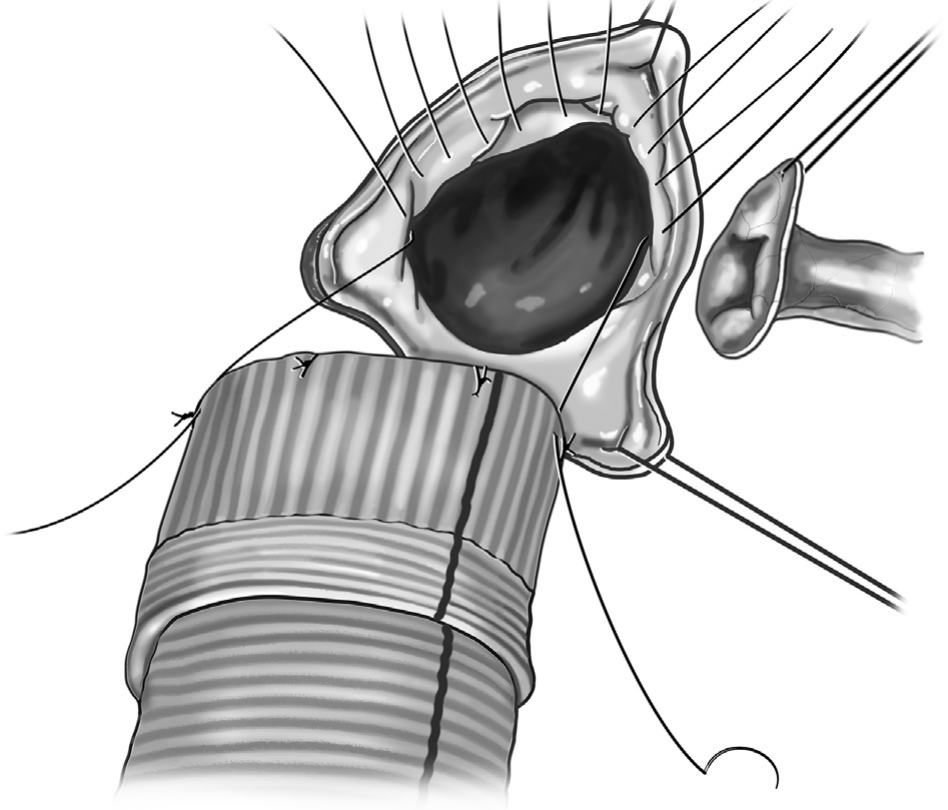
Autograft implantation. Native aortic annular sutures are placed circumferentially, planar, and evenly spaced. The first autograft suture is placed at the nadir of the identified sinus for left coronary button implantation. The next autograft suture is placed 180° from the initial one. Sutures are then placed circumferentially around the autograft and the neoaortic root is parachuted down.

The LVOT is prepared through the creation of coronary artery buttons and the excision of native aortic valve leaflets and ascending aorta just below the crossclamp. If the native aortic root is larger than the PA annulus, an inclusion technique is the preferred approach to implant the autograft. If the native aortic root is the same size or smaller than the PA annulus, then a free-standing approach can be used. For autograft implantation, simple interrupted sutures are used with 2-0 braided polyester suture (Figure 8). Sutures are first placed through the native aortic annulus, moving circumferentially. Annular sutures are kept planar and evenly spaced within the LVOT to avoid distortion of the autograft once implanted. Once all annular sutures are placed, the first suture is then placed into the autograft annulus at the nadir of the identified sinus for left coronary button implantation, ensuring to capture both the muscular cuff and root graft. Next, the suture that is located 180° from that initial coronary suture is placed into the autograft annulus. Sutures are then placed circumferentially around the autograft and the neoaortic root is parachuted down. Knots can be secured by hand tying or an automated knot-tying device. At this point, the valve can once again be tested by pressurization to ensure lack of distortion of the implanted autograft.

While still pressurized, the root graft is excised over the identified sinus for left coronary implantation, with care taken to avoid the previously placed commissural markings. As the pressurized autograft sinus bulges through the excised graft segment, an incision is made into the autograft and pressurization is stopped. The autograft sinus is trimmed minimally in order to avoid retraction of the autograft wall away from the root graft. Full-thickness bites are taken of the root graft, autograft, and coronary button wall using a running 5-0 monofilament suture. Once the left coronary is implanted, the distal anastomosis of the ascending graft is performed with running 4-0 monofilament suture. Similarly, the Dacron graft is trimmed while pressurized for the right coronary button anastomosis.

At this point, the crossclamp can be removed and the right ventricle (RV)-PA conduit distal anastomosis can be performed. The myocardial bed from autograft harvest is inspected for bleeding, and hemostasis is achieved. The proximal anastomosis of the RV-PA conduit is then performed. Subsequently, the distal end of the root reinforcing graft can be secured to the ascending aortic graft using 5-0 monofilament suture and simple interrupted sutures. CPB is discontinued and intraoperative postbypass transesophageal echocardiogram (TEE) is obtained to evaluate autograft performance.

### Postoperative Monitoring

In the immediate postoperative period, patients are administered medication for blood pressure control with goal systolic pressures less than 110 mm Hg. Because the autograft root is circumferentially reinforced, blood pressure control at home is less strict. Goal systolic pressure after discharge is less than 120 mm Hg, but we do not believe that aggressive antihypertensive management at home is necessary.

## Results

### Patient Demographics and Characteristics

Four patients underwent modified reinforced Ross with preimplantation root pressurization between September 2024 and January 2025. Two patients were female, the median age of the cohort was 47.5 (27-55) years, weight at surgery was 79.5 (57-90) kg, and body mass index was 26 (23-29) kg/m^2^. The primary diagnosis was aortic stenosis for 3 patients and aortic regurgitation for 1 patient. All 4 patients had ascending aorta dilation. Native aortic valve morphology was bicuspid for 3 patients and unicuspid for 1 patient. One patient previously underwent balloon dilation; no other patients had previous cardiac interventions.

### Preoperative Measurements and Operative Details

On preoperative computed tomography scan, the native pulmonary annulus diameter of all 4 patients ranged from 23 to 31 mm. Native aortic diameters ranged from 20 to 33 mm for aortic annulus and 36 to 51.5 mm for ascending aorta. Full valve and graft measurements are described in Table 1.

**Table 1:** Perioperative measurements and Dacron graft sizing

Measured values	Patient 1	Patient 2	Patient 3	Patient 4
Preoperative measurements				
BSA, m^2^	1.87	2.07	2.11	1.58
Aortic valve				
Ascending aorta diameter,∗ mm	36	51.5	46	49
Annulus diameter,∗ mm	25	29	33	20
Regurgitation	None	None	Severe	Mild/moderate
Stenosis	Severe	Severe	Mild/moderate	Severe
Pulmonary valve				
Annulus diameter,∗ mm	23	28	31	26
Regurgitation	None	None	Trivial	Trivial
Stenosis	None	None	None	None
Intraoperative Dacron graft sizing, mm				
Ascending aorta	22	28	28	26
Root reinforcing (Valsalva)	26	28	32	28
Intraoperative measurements				
Neoaortic valve				
Regurgitation	Trivial	None	Trivial	Trivial
Stenosis	None	None	None	None

*BSA*, Body surface area.

∗ Measured on cross-sectional imaging.

Pulmonary root harvest was done under crossclamp for 3 patients and on bypass with beating heart for 1 patient. Median bypass time was 201 minutes (193-230 minutes), and crossclamp time was 167.5 minutes (149-175 minutes). All procedures were completed with 2 attending surgeons, with one preparing the aortic root while the other prepared the Ross autograft.

Modifications to the Ross autograft facilitated by back table pressurization included trimming of muscular cuff and adventitial fat in all 4 patients, a subcommissural plication stitch in one patient (which significantly improved autograft regurgitation), Sinus of Valsalva root reinforcing graft placement with optimized leaflet/commissural orientation in all 4 patients, and root graft/autograft plication in one patient.

### Postoperative Measurements and Outcomes

After CPB was discontinued, Ross autograft function was measured with intraoperative TEE which demonstrated neoaortic regurgitation levels of none for 1 patient and trivial for 3 patients (Table 1). Neoaortic stenosis was none for all four patients. There was 1 patient with mild left ventricular (LV) dysfunction and trivial pulmonary homograft regurgitation. All other patients had no LV dysfunction or pulmonary homograft regurgitation. No patients had LV dilation or RV dysfunction/dilation.

Median systolic blood pressure was 116 mm Hg (range, 103-128 mm Hg) on arrival to the cardiac ICU. Two patients were placed on norepinephrine for blood pressure optimization, 1 patient was placed on nicardipine, and 1 patient was not on any vasoactives. Median ICU length of stay was 2.5 days (1-5 days), and hospital length of stay was 8.5 days (8-12 days). No patients had postoperative complications including infection, arrhythmia/heart block, renal injury, liver injury, stroke, seizure, re-exploration, reinterventions, or mechanical circulatory support, monitored through 30 days after discharge. There were no mortalities. Median systolic blood pressure was 100 mm Hg (97-139 mm Hg) at discharge, and all patients required 1-3 anti-hypertensive medications. Antihypertensives included amlodipine, labetalol, lisinopril, metoprolol, and valsartan. Three patients were discharged home, and 1 patient was discharged to a rehabilitation center because of a preexisting diagnosis affecting physical mobility.

## Discussion

To our knowledge, this is the first description of a surgical technique to assess and modify valvular function under pressurized conditions before Ross autograft implantation. This technique allows for optimization of postoperative autograft function through patient-specific modifications, which may reduce variability in outcomes and enhance the longevity of the reinforced Ross. Pressurization of the pulmonary autograft facilitates these proposed benefits by allowing for the following two unique vantage points:

1. Direct inspection of leaflet coaptation from the ventricular side. This allows the degree and location of regurgitation to be directly visualized. If a deficient area is identified, the surgeon can temporarily manipulate the autograft root, watch the effect of that manipulation in real time, and select the most effective location to provide additional support. For example, upon direct inspection of leaflet coaptation for our second patient, it was evident that there was mild-to-moderate regurgitation at the coaptation zone between two leaflet cusps along a shared commissure. A subcommissural plication stitch was placed, and, upon repressurization, there was no longer regurgitation at this location. This patient was taken off bypass with no autograft regurgitation.
2. Visualization of the pressurized autograft within its reinforcing root graft. This allows for optimal fitting of the autograft within its reinforcing graft. If the autograft appears too crowded once fully pressurized, additional adventitial fat and muscular trimming can be performed. Conversely, if while pressurized, it is evident that the autograft needs more support, the Dacron graft can be plicated to reduce the autograft root size. Alteration of the root can be done while pressurized to watch the effects of size reduction on degree of regurgitation, allowing for identification of optimal graft sizing. In addition, this visualization allows for informed manipulation to find the ideal plane and height at which the valve sits within the graft. Once this plane is identified, the reinforcing graft can be secured while still pressurized to ensure the orientation is maintained.

The Ross procedure has evolved considerably since its advent more than 50 years ago. Contemporary techniques such as root graft reinforcement have addressed initial concerns for greater reintervention rates, and tailored approaches continue to improve long-term valve function and late survival.^12^ Our reproducible technique of Ross autograft pressurization builds upon this work and allows for a greater level of personalized modification to both the autograft and root graft.

Many of these optimization techniques are an established part of the Ross surgeon's armamentarium when unfavorable autograft function is found on postoperative TEE. In these instances, pre-implantation pressurization may help prevent repeat bypass runs; as potential issues were already prophylactically addressed with familiar repair techniques under ideal visualization conditions.

There are several ways of performing autograft root reinforcement. Our center prefers circumferential reinforcement because it prevents dilation along the entire length of autograft and facilitates less annular variability after implantation. Securing the Dacron to the autograft skirt while the root is pressurized helps minimize distortion of the autograft commissures and sinuses during the proximal anastomosis. In addition, the Dacron reinforcement along the proximal suture line stabilizes the root significantly, allowing the majority of the load to be borne by the Dacron, consequently preserving valvular function and protecting against dehiscence. This level of reinforcement may also be an advantage for patients with hypertension, as it may allow for less strict systolic blood pressure control long term. It is currently unknown whether circumferential reinforcement is absolutely necessary or whether there are long-term disadvantages to this technique. Of note, root stabilization with a Dacron graft limits the autograft's growth potential, an important consideration for the pediatric population.

Another concern with circumferential reinforcement is the hemodynamic consequence of compromised root elasticity.^13^ For this reason, we prefer Valsalva shaped compliant grafts as our root-reinforcing graft, selected at a nonrestricting size. The Valsalva graft diameter is typically 4 mm larger than the preoperative measurement of the PA sinotubular junction diameter. This accounts for 1 to 2 mm of autograft wall thickness, as well as the average 1 to 2 mm of aortic root expansion during the cardiac cycle.^14^^,^^15^ Valve-sparing aortic root replacements using Valsalva grafts have had favorable mid- and long-term follow-up,^16^^,^^17^ although data for Valsalva graft use in the Ross procedure are lacking.

Our pressurization technique has developed over time. We have previously used saline for pressurization in porcine experiments but found that the distal graft suture line displayed high levels of leakage and, therefore, we prefer whole blood as the medium for pressurization. This method also allows for evaluation of bleeding at the distal suture line. Because valve tissue has the ability to diffuse oxygen directly, it may also be beneficial for the valve leaflet endothelium to be exposed to whole blood during the preparation process.

An additional benefit to pressurizing the Ross autograft is the ability to excise the point for coronary entry with the leaflets away from the sinus wall. Our technique for coronary button implantation allows for better stabilization of the coronary anastomosis by involving the Dacron graft. To avoid hematoma formation between the autograft and root-reinforcing graft, the distal end of the reinforcing graft is secured in an interrupted fashion with adequate space between sutures to allow collected blood to escape.

### Limitations

Currently at our center, we are using the autograft pressurization technique for all adult patients with aortic root size greater than 20 mm undergoing the Ross procedure. Future work to establish predictive factors for autograft optimization procedures would be beneficial in identifying the optimal population for this technique, although we expect the greatest benefit will be to those with aortic insufficiency before operation. Another limitation to this technique is its current lack of long-term data, and the consequent inability to correlate autograft function before implantation with echocardiographic outcomes. Further data for this patient cohort will continue to be collected, with particular attention to autograft dilation and dysfunction at mid- and late-term follow-up. In addition, this technique is most efficient when 2 surgeons work simultaneously to optimize the Ross autograft and prepare the native aortic root. However, autograft optimization is still feasible with a single surgeon. As compared with the traditional reinforced Ross, the only additional steps are the evaluation of the autograft under pressurized conditions and the associated optimization procedures such as plications. Even with a single surgeon, this still produces an acceptable crossclamp time, especially in the setting of pulmonary root harvest on bypass with beating heart, as is our center's preference.

## Conclusions

The pressurization technique of the reinforced Ross pulmonary autograft allows for optimization of autograft valve performance and annular/root stabilization before implantation. The vantage points created through pressurization allow the surgeon to perform individualized supportive procedures that may enhance autograft function in the short and long term.

### Webcast

You can watch a Webcast of this AATS meeting presentation by going to: Xxx.

## Conflict of Interest Statement

The authors reported no conflicts of interest.

The *Journal* policy requires editors and reviewers to disclose conflicts of interest and to decline handling or reviewing manuscripts for which they may have a conflict of interest. The editors and reviewers of this article have no conflicts of interest.

## Supplementary Data

Video 1 Ross autograft root pressurization before implantation. Video available at: https://www.jtcvs.org/article/S2666-2507(25)00138-5/fulltext.

## References

[1] El-Hamamsy I., Eryigit Z., Stevens L.M. (2010). Long-term outcomes after autograft versus homograft aortic root replacement in adults with aortic valve disease: a randomised controlled trial. Lancet.

[2] El -Hamamsy I., Toyoda N., Itagaki S. (2022). Propensity-matched comparison of the ross procedure and prosthetic aortic valve replacement in adults. J Am Coll Cardiol.

[3] Mazine A., David T.E., Stoklosa K., Chung J., Lafreniere-Roula M., Ouzounian M. (2022). Improved outcomes following the Ross procedure compared with bioprosthetic aortic valve replacement. J Am Coll Cardiol.

[4] Mazine A., Rocha R.V., El-Hamamsy I. (2018). Ross procedure vs mechanical aortic valve replacement in adults: a systematic review and meta-analysis. JAMA Cardiol.

[5] Mazine A., David T.E., Rao V. (2016). Long-term outcomes of the Ross procedure versus mechanical aortic valve replacement. Circulation.

[6] Sherif N.E., Dearani J.A., Connolly H.M. (2023). Complexity and outcome of reoperations after the Ross procedure in the current era. Ann Thorac Surg.

[7] Stulak J.M., Burkhart H.M., Sundt T.M. (2010). Spectrum and outcome of reoperations after the Ross procedure. Circulation.

[8] Jacobsen R.M., Earing M.G., Hill G.D. (2015). The externally supported Ross operation: early outcomes and intermediate follow-up. Ann Thorac Surg.

[9] Bansal N., Kumar S.R., Baker C.J., Lemus R., Wells W.J., Starnes V.A. (2015). Age-related outcomes of the Ross procedure over 20 years. Ann Thorac Surg.

[10] Starnes V.A., Elsayed R.S., Cohen R.G. (2023). Long-term outcomes with the pulmonary autograft inclusion technique in adults with bicuspid aortic valves undergoing the Ross procedure. J Thorac Cardiovasc Surg.

[11] Caldaroni F., Skillington P., O’Keefe M., Buratto E., Wynne R. (2025). 25 years of Ross operation in adults: the inclusion technique keeps up the expectations. J Thorac Cardiovasc Surg.

[12] Bouhout I., Chauvette V., Notenboom M.L., Poirier N., Demers P., El-Hamamsy I. (2024). Abstract 4141165: long-term contemporary outcomes of the Ross procedure. Circulation.

[13] Tanaka D., Mazine A., Ouzounian M., El-Hamamsy I. (2022). Supporting the Ross procedure: preserving root physiology while mitigating autograft dilatation. Curr Opin Cardiol.

[14] Lansac E., Lim H.S., Shomura Y. (2002). A four-dimensional study of the aortic root dynamics. Eur J Cardiothorac Surg.

[15] Blanke P., Russe M., Leipsic J. (2012). Conformational pulsatile changes of the aortic annulus: impact on prosthesis sizing by computed tomography for transcatheter aortic valve replacement. JACC Cardiovasc Interv.

[16] De Paulis R., Scaffa R., Nardella S. (2010). Use of the Valsalva graft and long-term follow-up. J Thorac Cardiovasc Surg.

[17] De Paulis R., Chirichilli I., Scaffa R. (2016). Long-term results of the valve reimplantation technique using a graft with sinuses. J Thorac Cardiovasc Surg.

