# Supplementary figures and images for: A modification of the reinforced Ross procedure: Root pressurization before implantation
Source: JTCVS Tech. 2025 May 4;31:108–14. doi: 10.1016/j.xjtc.2025.03.017 (PMC12237868; doi:10.1016/j.xjtc.2025.03.017)

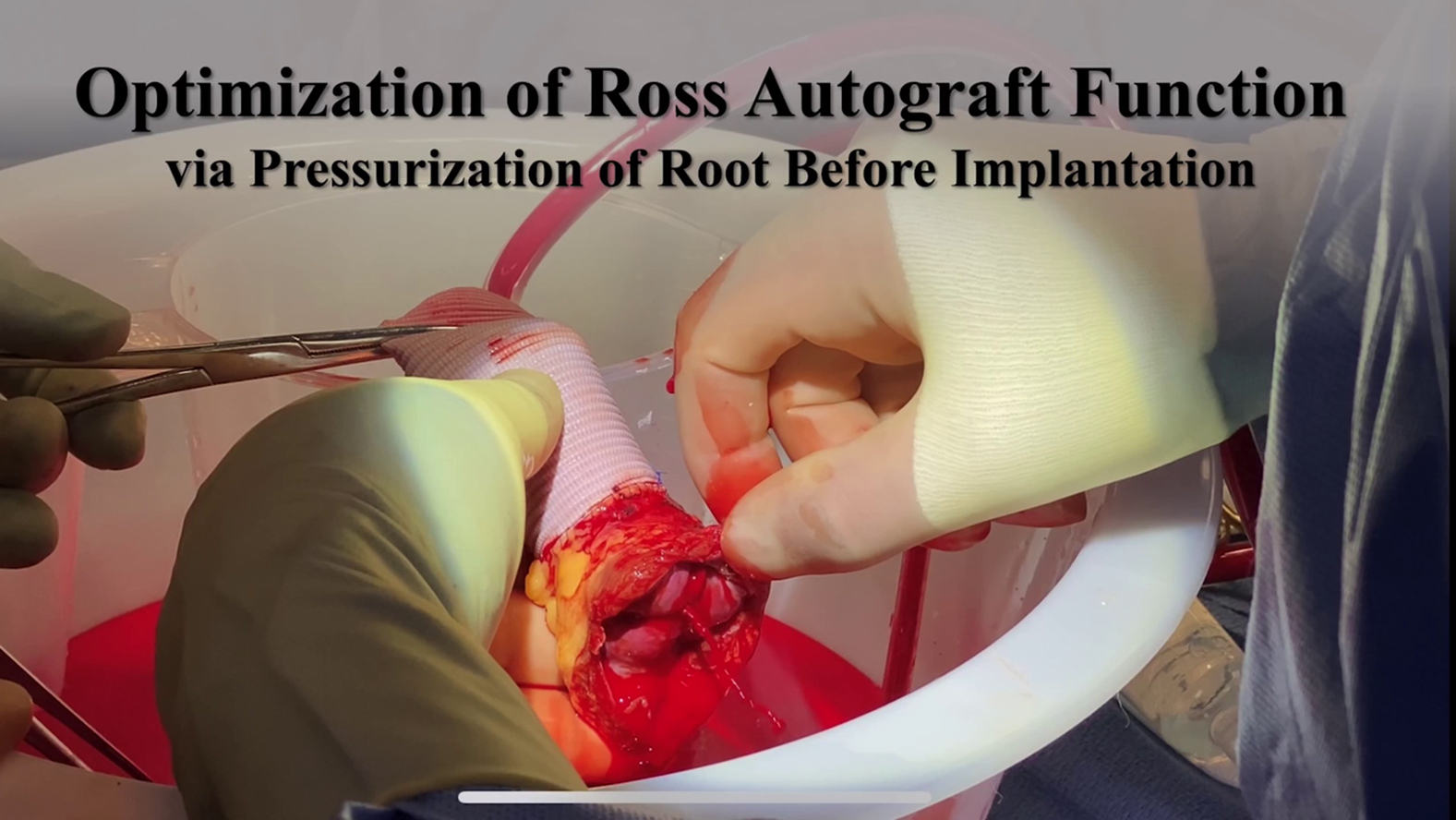

Supplement: Video 1 — Ross autograft root pressurization before implantation. Video available at: https://www.jtcvs.org/article/S2666-2507(25)00138-5/fulltext. [file fx2.jpg]
